# Impact of *Helicobacter pylori* Infection and Its Major Virulence Factor CagA on DNA Damage Repair

**DOI:** 10.3390/microorganisms8122007

**Published:** 2020-12-16

**Authors:** Eleftherios Kontizas, Spyros Tastsoglou, Timokratis Karamitros, Yiannis Karayiannis, Panagoula Kollia, Artemis G. Hatzigeorgiou, Dionyssios N. Sgouras

**Affiliations:** 1 Laboratory of Medical Microbiology, Hellenic Pasteur Institute, 11521 Athens, Greece; jkaragiannis@pasteur.gr; 2 Department of Genetics and Biotechnology, Faculty of Biology, National and Kapodistrian University of Athens, 15772 Athens, Greece; pankollia@biol.uoa.gr; 3 Department of Electrical and Computer Engineering, University of Thessaly, 38221 Volos, Greece; tastsoglou@uth.gr; 4 DIANA-Lab, Hellenic Pasteur Institute, 11521 Athens, Greece; arhatzig@inf.uth.gr; 5 Bioinformatics and Applied Genomics Unit, Hellenic Pasteur Institute, 11521 Athens, Greece; tkaram@pasteur.gr; 6 DIANA-Lab, Department of Computer Science and Biomedical Informatics, University of Thessaly, 35131 Lamia, Greece

**Keywords:** *Helicobacter pylori*, CagA, DNA damage repair, gastric carcinogenesis

## Abstract

*Helicobacter pylori* infection induces a plethora of DNA damages. Gastric epithelial cells, in order to maintain genomic integrity, require an integrous DNA damage repair (DDR) machinery, which, however, is reported to be modulated by the infection. CagA is a major *H. pylori* virulence factor, associated with increased risk for gastric carcinogenesis. Its pathogenic activity is partly regulated by phosphorylation on EPIYA motifs. Our aim was to identify effects of *H. pylori* infection and CagA on DDR, investigating the transcriptome of AGS cells, infected with wild-type, ΔCagA and EPIYA-phosphorylation-defective strains. Upon RNA-Seq-based transcriptomic analysis, we observed that a notable number of DDR genes were found deregulated during the infection, potentially resulting to base excision repair and mismatch repair compromise and an intricate deregulation of nucleotide excision repair, homologous recombination and non-homologous end-joining. Transcriptome observations were further investigated on the protein expression level, utilizing infections of AGS and GES-1 cells. We observed that CagA contributed to the downregulation of Nth Like DNA Glycosylase 1 (NTHL1), MutY DNA Glycosylase (MUTYH), Flap Structure-Specific Endonuclease 1 (FEN1), RAD51 Recombinase, DNA Polymerase Delta Catalytic Subunit (POLD1), and DNA Ligase 1 (LIG1) and, contrary to transcriptome results, Apurinic/Apyrimidinic Endodeoxyribonuclease 1 (APE1) upregulation. Our study accentuates the role of CagA as a significant contributor of *H. pylori* infection-mediated DDR modulation, potentially disrupting the balance between DNA damage and repair, thus favoring genomic instability and carcinogenesis.

## 1. Introduction

Gastric cancer is the fifth most common type of cancer and the third most prevalent cause of cancer death worldwide [1]. The best established risk factor for gastric carcinogenesis is *Helicobacter pylori* infection [2,3], particularly associated with gastric adenocarcinoma and mucosa-associated lymphoid tissue (MALT) lymphoma development [4,5,6,7]. *H. pylori* infection can promote chronic inflammation by inducing gradual mucosal alterations that ultimately can lead to gastric adenocarcinoma [8]. The risk of gastric cancer development is determined by specific interactions between *H. pylori* and the host, which in turn are dependent on the expression of strain-specific bacterial virulence factors [9], inducing a variable inflammatory response governed by host genetic predisposition, as well as environmental factors [10,11].

A major virulence factor of *H. pylori* is the CagA protein. CagA is encoded by *cag* pathogenicity island (*cag*PAI) and is translocated intracellularly, via the type IV secretion system (T4SS) [12]. Its pathogenic activity is partly regulated via hierarchic tyrosine phosphorylation by kinases of the host gastric epithelial cells, at repeating Glu–Pro–Ile–Tyr–Ala (EPIYA) motifs, located at the C-terminus of the protein [13,14]. EPIYA sequence motifs can be classified as EPIYA-A, EPIYA-B, EPIYA-C, and EPIYA-D motifs, depending on their surrounding sequence [15]. In *H. pylori* strains derived from Western countries, EPIYA-A and EPIYA-B motifs have been reported, typically followed by one to four copies of EPIYA-C, whereas the combination of EPIYA-A and EPIYA-B with single EPIYA-D motifs has been predominantly identified in strains isolated in East Asia. Upon its delivery to the cytoplasm, CagA can interact, in an EPIYA-phosphorylation-dependent or -independent manner, with several host proteins, thus deregulating crucial cellular functions such as proliferation, apoptosis, inflammation, and genomic integrity [16,17,18]. Infection with CagA-positive strains has been reported to augment inflammation in the gastric mucosa, triggering oxidative stress and DNA damage, thus favoring genomic instability and increasing the risk for gastric cancer development [19,20,21].

*H. pylori* infection can induce chronic active inflammation and a plethora of DNA damages predisposing to genomic instability. More specifically, the infection and CagA positivity have been shown to increase levels of reactive oxygen and nitrogen species (RONS), which can lead to the formation of oxidative DNA damage, most notably generation of 8-oxo-guanine (8-oxo-G) and apurinic/apyrimidinic sites (AP-sites), as well as DNA strand breaks and bulky DNA adducts [21,22,23,24,25]. One characteristic type of DNA damage reported during *H. pylori* infection is the formation of double-strand breaks (DSBs) [23,26,27,28,29], which accumulate in actively transcribed and telomere-proximal chromosome regions [30]. The occurring DSBs can be introduced not only as a consequence of the inflammation-induced oxidative and replication stress, but also as a result of direct bacterial attachment to the host cells [26,28]. *Cag*PAI-positive *H. pylori* strains have been reported to activate NF-κB signaling resulting to aberrant expression of Activation-Induced Cytidine Deaminase (AID), which can subsequently induce nucleotide alterations, including *TP53* mutations and potential introduction of DSBs [31,32]. In addition, CagA has been shown to interact with and inhibit Partitioning-defective 1 (PAR1)/Microtubule Affinity-Regulating Kinase (MARK), thus promoting microtubule malfunction, DSB introduction, and chromosomal instability [33,34]. *H. pylori* infection has also been reported to induce microsatellite instability (MSI), as well as frameshift and point mutations [31,35,36,37,38]. Apart from nuclear DNA, the infection also promotes mutations in mitochondrial DNA (mtDNA), including MSI [39] and base substitutions, of which transitions are reported to be the main mutational event and significantly increased during infection with strains expressing CagA and VacA virulence factors [35,40]. Another important impact of *H. pylori* infection on DNA integrity is the hypermethylation of the promoter regions of numerous genes, e.g., *MLH1*, *CDH1*, and *MGMT*, leading to their silencing [41].

Human cells have evolved a sophisticated network of cellular pathways, termed DNA damage response, that prevents genomic instability and its biological consequences. This complex network includes diverse and coordinated cellular functions such as transcription, cell-cycle checkpoints, DNA damage repair (DDR), and cell death [42]. Base excision repair (BER), nucleotide excision repair (NER), mismatch repair (MMR), homologous recombination (HR), and non-homologous end-joining (NHEJ) are the major DDR mechanisms that human cells utilize in order to maintain genome integrity [43].

The emerging DNA damages in gastric cells during *H. pylori* infection necessitate the activation of DDR mechanisms, which in turn have been reported to be modulated by the infection [44]. One of the most critical effects of *H. pylori* infection is the CagA-mediated degradation of TP53 via proteasome [45,46,47]. In addition, key components of genome maintenance have been reported to show decreased expression or activation during infection, including *MGMT* methyltransferase, which has been reported to be silenced via promoter hypermethylation [48]. Furthermore, the infection has been reported to downregulate MMR components, including MutL Homolog 1 (MLH1), MutS Homolog 2 (MSH2), MSH3, MSH6, Post-Meiotic Segregation Increased 1 (PMS1), PMS2, and DNA Polymerase Delta Subunit 3 (POLD3) [35,49,50,51]. APE1 is a central component of BER which has been reported to be upregulated during the infection [52,53], whereas other studies support its downregulation [35]. *H. pylori* infection promotes the formation of DSBs, which activate DDR mechanisms that can efficiently repair the emerging damages during short-term infection. However, prolonged infection and high multiplicity of infection (MOI) can saturate DDR, leading to damage accumulation [26,27,30]. With this regard, the infection has been reported to attenuate the expression or activation of significant factors related to DSBs repair, including Meiotic Recombination 11 Homolog A (MRE11), Nijmegen Breakage Syndrome 1 (NBS1), Ataxia Telangiectasia and Rad3-Related Protein (ATR), ATR-Interacting Protein (ATRIP), RAD51 and RAD54L with a more prominent modulation of DDR observed in the infection with *cag*PAI-positive compared to *cag*PAI-negative strains [27,30]. Despite the reported modulation of DDR by the pathogen, the underlying mechanisms are still under investigation.

Our study aimed to characterize the impact of *H. pylori* infection on DDR mechanisms and attempted to identify putative underlying mechanisms perturbed by CagA and its intracellular phosphorylation. To this end, we performed RNA-Seq-based transcriptomic analysis on AGS cells in vitro infected with the P12 *H. pylori* strain, its corresponding ΔCagA mutant, and its isogenic CagA phosphorylation-defective mutant [54]. Key DDR components that were observed to be deregulated were further investigated at the protein expression level, in infected AGS and GES-1 gastric epithelial cell lines. Our data suggest that *H. pylori* infection can transcriptionally deregulate a notable number of DDR components of gastric cells, out of which the overwhelming majority was downregulated, indicating an attenuation of BER and MMR and a more intricate deregulation of NER, HR, and NHEJ. Furthermore, the downregulation of *NTHL1*, *MUTYH*, *FEN1*, *RAD51*, *POLD1*, and *LIG1* was verified at the protein level, highlighting the contribution of CagA in their downregulation. APE1 protein levels were also found to be increased, potentially due to CagA expression. These results suggest that CagA can act as a significant compromising factor of DDR, which could favor genomic instability in gastric epithelial cells, via putative disruption of the equilibrium between DNA damage introduction and repair, thus increasing the risk for gastric cancer development.

## 2. Materials and Methods

### 2.1. H. pylori and Gastric Epithelial Cell Culture

Wild-type P12 reference strain (NC_011498), a kind gift by Prof. R. Haas (Ludwig Maximilian University of Munich), is a Western-type *H. pylori* expressing CagA protein containing EPIYA-ABCC motifs (hereafter indicated as ABCC). Its corresponding ΔCagA strain (hereafter indicated as ΔCagA) and the isogenic phosphorylation-deficient counterpart that expresses CagA containing EPIFA motifs, following tyrosine substitution by phenylalanine in the terminal EPIYA-C domains (hereafter indicated as ABFF), were generated as described earlier [54,55]. These strains have been meticulously evaluated for the absence of potential polar effects, having been shown to grow with comparable rates, to adhere equally well to gastric epithelial cells, to induce pilus formation, and to express similar levels of CagA protein, which is functionally translocated and phosphorylated within epithelial cells [54,55,56]. Bacterial strains were cultured on Columbia Blood Agar (Oxoid, Nepean, ON, Canada) including antibiotics, supplemented with 5% *v/v* horse blood and 1% *v/v* Vitox (Oxoid), under microaerophilic conditions (CampyGen, Oxoid) at 37 °C, as previously described [54].

Human gastric epithelial cell lines AGS (ATCC CRL-1739) and GES-1 cells (kindly provided by Dr. D. Kidane, University of Texas at Austin) were cultured in 75 cm^2^ flasks (Corning, NY, USA) in RPMI-1640 medium (Gibco, Grand Island, NY, USA), supplemented with 10% FBS (Gibco), including antibiotics (penicillin 10 U/mL, streptomycin 10 mg/mL), at 37 °C in a humidified chamber supplemented with 5% CO_2_.

### 2.2. In Vitro Infection of Gastric Epithelial Cells

Gastric epithelial cells were infected with *H. pylori* at a MOI of 100, as previously described [55]. Briefly, 4 × 10^5^ cells were seeded in six-well plates (Corning) and were left to adhere and grow overnight. Two hours prior to infection, cells were rinsed twice with PBS (1×) and the medium was replaced with 2 mL of antibiotic-free RPMI-1640 supplemented with 10% FBS. Bacterial strains were suspended within the same medium and 100 μL suspensions were utilized to infect the cells.

### 2.3. RNA Isolation, RNA-Seq, and Bioinformatics

Total RNA was extracted from 24 h *H. pylori*-infected and uninfected AGS cells, using TRIzol Reagent (Invitrogen, Carlsbad, CA, USA) and purified utilizing DNAaseI (Promega, Madison, WI, USA) and RNAeasy Mini kit (Qiagen, Hilden, Germany). PolyA-tailed transcripts were purified using a Dynabeads messenger RNA (mRNA) DIRECT Micro Kit (Thermo Fisher Scientific, Waltham, MA, USA) and sequencing libraries were prepared using Ion Total RNA-Seq Kit v2 according to the manufacturer’s protocol (Thermo Fisher Scientific). Template preparation was implemented on Ion Chef using Ion PI Hi-Q OT2 200 Kit and sequencing was performed on Ion PI Hi-Q Sequencing 200 Kit utilizing Ion Proton PI chips (Thermo Fisher Scientific), according to the manufacturer’s protocol.

RNA-Seq data were registered in the Gene Expression Omnibus (GEO) with accession number GSE162056. FASTQ files were cleared from adapters and low-quality reads [57] and aligned on the human genome assembly hg19 using TopHat2 v2.2.1 at default settings and Illumina iGenomes transcript annotation (UCSC hg19 build, Illumina, San Diego, CA, USA) [58]. Unaligned reads were re-aligned on hg19 using Bowtie2 v2.2.7 (parameter “very-sensitive”) and merged with initial alignments. Gene counts were estimated applying GenomicRanges [59], while the metaseqR tool v1.10.0 [60] was employed for quality control filtering and differential expression analysis. Genes were filtered for length, uniformity of coverage, and total counts utilizing metaseqR default *gene.filters* options. Bioconductor package edgeR v3.12.1 was utilized for the normalization of gene counts and differential expression analysis [61]. We compared the counts from ABCC-infected AGS vs. uninfected AGS, to investigate the role of *H. pylori* infection and the ABCC-infected AGS vs. ΔCagA-infected AGS to predict the putative role of CagA protein. Lastly, a comparison between ABCC-infected AGS vs. ABFF-infected AGS, was utilized to explore potential role of CagA phosphorylation at EPIYA-C motifs. Genes presenting |log2(FoldChange)| > 0.5 and false discovery rate (FDR) < 0.05 were considered as significantly differentially expressed and were subjected to pathway enrichment analysis that focused on the DDR KEGG pathways, applying kegga() function from limma package v3.38.3 [62,63,64]. DDR pathways fulfilling the threshold of FDR < 0.05 were considered as significantly enriched. Visualization of pathway enrichment results was conducted with Pathview R package v1.22.3 [65]. The heatmap for DDR genes that were differentially expressed in any of the investigated comparisons was created using the normalized gene counts.

### 2.4. Determination of Protein Expression Levels by Western Blot Analysis

Whole-cell lysates from 24 h infected AGS and GES-1 cells were obtained in ice-cold radioimmunoprecipitation assay buffer (RIPA) supplemented with protease and phosphatase inhibitors, as previously described [54]. Total protein concentration was determined via Pierce BCA Protein Assay Kit (Thermo Fisher Scientific) and lysates were separated by SDS-PAGE and transferred onto Immobilon PVDF membranes (Merck Millipore, Darmstadt, Germany). Western blots were performed utilizing antibodies against Ser139 phosphorylated histone H2AX (γH2AX) (Cell Signaling, Danvers, MA, USA), RAD51 (Cell Signaling), APE1 (Novus Biologicals, Centennial, CO, USA), LIG1 (Novus Biologicals), NTHL1 (Santa Cruz, Dallas, TX, USA), MUTYH (Santa Cruz), FEN1 (Santa Cruz), POLD1 (Santa Cruz), and GAPDH (Santa Cruz).

## 3. Results

**Figure 1:**
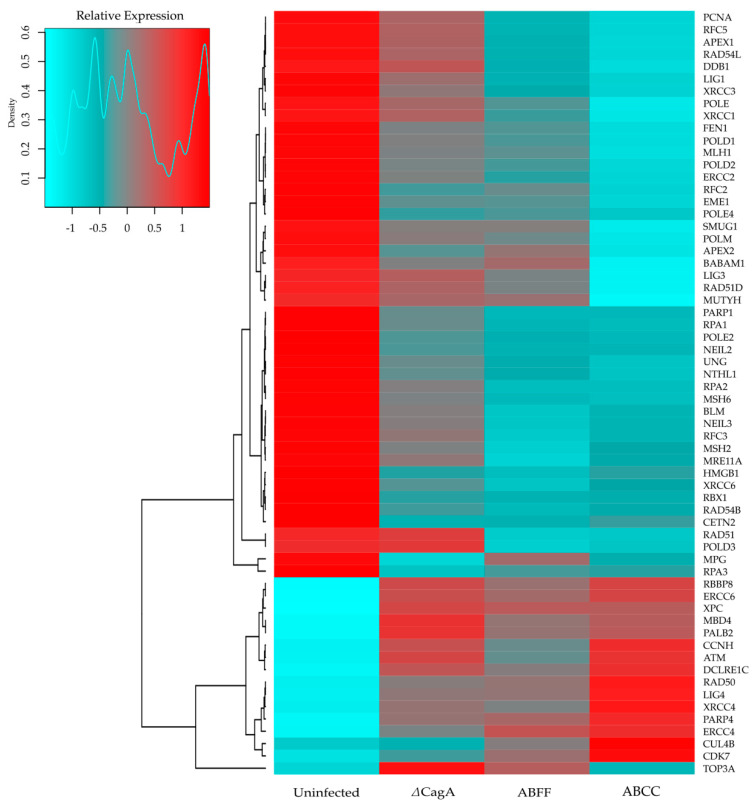
Gene expression profile related to DNA damage repair in the context of *H. pylori* infection. RNA-Seq analysis was performed on AGS cells infected with *H. pylori* P12 ABCC, ABFF, and ΔCagA strains.

Differential expression analysis showed that *H. pylori* infection on AGS cells deregulated the transcription of 5227 genes, of which 2640 were upregulated and 2587 were downregulated. CagA contributed to the deregulation of 657 genes, of which 344 were upregulated and 313 were downregulated. Putative EPIYA-C phosphorylation of CagA was determined to be involved in the deregulation of 21 genes, of which nine were upregulated and 12 were downregulated. Pathway enrichment analysis indicated that *H. pylori* infection can potentially affect every major DDR mechanism and CagA protein can be a significant contributor to the deregulation of BER, NER, MMR, and HR mechanisms (Table 1), whereas CagA phosphorylation is suggested not to have a significant impact on the deregulation of those mechanisms. Furthermore, via heatmap clustering, we visualized and identified expression patterns of clusters of genes involved in DDR, relating to *H. pylori* infection, as well as the expression and phosphorylation of CagA protein (Figure 1).

### 3.1. Base Excision Repair

**Figure 2:**
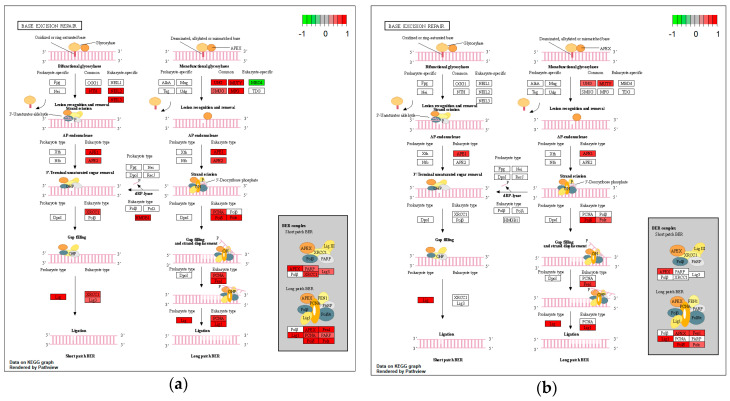
Impact of *H. pylori* infection (**a**) and CagA protein (**b**) on gene expression of base excision repair (BER) components, visualized in KEGG pathway map. Components involved in the process are highlighted in red (downregulated) or green (upregulated).

Of the 33 genes involved in BER, 24 were found to be deregulated upon *H. pylori* infection (Figure 2a), nine of which were predicted to be in a CagA-related manner (Figure 2b). More specifically, with regard to *H. pylori* infection, 22 BER components were found downregulated, including glycosylases (*NTHL1*, *MUTYH*, *UNG*, *NEIL2*, *NEIL3*, *MPG*, and *SMUG1*), polymerases (*POLD1*, *POLD2*, *POLD3*, *POLE*, *POLE2*, and *POLE4*), ligases (*LIG1* and *LIG3*), and endonucleases (*APE1*, *APE2*, and *FEN1*), as well as *PCNA*, *XRCC1*, *HMGB1* and *PARP1* (Figure 2a). On the contrary two components were found upregulated, namely, *MBD4* glycosylase and *PARP4* polymerase. CagA expression appeared to contribute to the downregulation of nine genes (Figure 2b), namely, *UNG* and *MUTYH* glycosylases, *APE1* and *FEN1* endonucleases, *POLD1*, *POLD2*, *POLD3*, and *POLE* polymerases, and *LIG1* ligase.

### 3.2. Nucleotide Excision Repair

**Figure 3:**
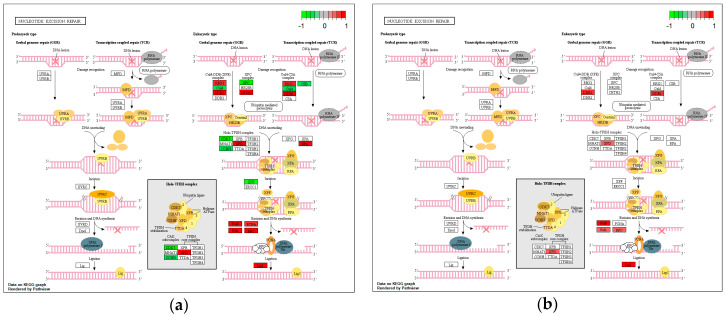
Impact of *H. pylori* infection (**a**) and CagA protein (**b**) on gene expression of nucleotide excision repair (NER) components, visualized in KEGG pathway map. Components involved in the process are highlighted in red (downregulated) or green (upregulated).

Of the 47 genes involved in NER, 24 were found differentially expressed upon *H. pylori* infection (Figure 3a). Specifically, genes *XPC*, *CUL4B*, and *ERCC6* (*CSB*), involved in damage recognition, and three genes (*CDK7*, *CCNH*, *XPF*), mediating DNA unwinding and incision were found upregulated. In addition, 18 genes were found downregulated, namely, three genes (*RBX1*, *CETN2*, *DDB1*) involved in damage recognition, four genes (*XPD*, *RPA1*, *RPA2*, *RPA3*) involved in DNA unwinding and incision, and 11 genes (*PCNA*, *RFC2*, *RFC3*, *RFC5*, *POLD1*, *POLD2*, *POLD3*, *POLE*, *POLE2*, *POLE4*, *LIG1*) mediating DNA synthesis and ligation. CagA protein appeared to contribute to the downregulation of eight out of the aforementioned genes, namely, *DDB1*, *XPD*, *RFC5*, *POLD1*, *POLD2*, *POLD3*, *POLE*, and *LIG1* (Figure 3b).

### 3.3. Mismatch Repair

**Figure 4:**
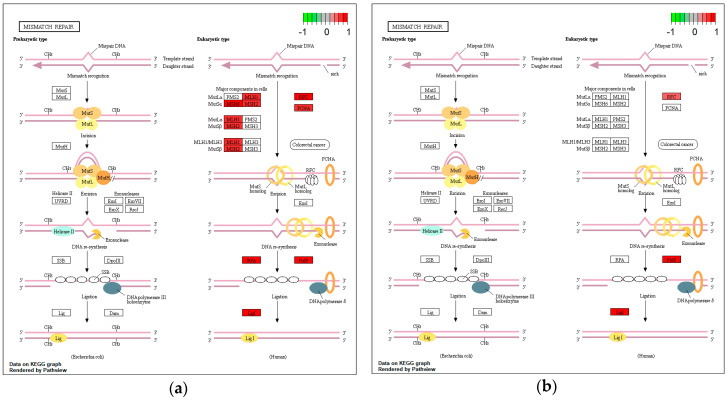
Impact of *H. pylori* infection (**a**) and CagA protein (**b**) on gene expression of mismatch repair (MMR) components, visualized in KEGG pathway map. Components involved in the process are highlighted in red (downregulated) or green (upregulated).

*H. pylori* infection resulted in the downregulation of key genes mediating MMR such as *MLH1*, *MSH2*, and *MSH6*, involved in damage recognition and excision, *POLD1*, *POLD2*, *POLD3*, and *LIG1*, mediating DNA synthesis and ligation, and the accessory components *RPA1*, *RPA2*, *RPA3*, *RFC2*, *RFC3*, *RFC5*, and *PCNA* (Figure 4a). The downregulation of *POLD1*, *POLD2*, *POLD3, RFC5*, and *LIG1* was predicted to be CagA-related (Figure 4b).

### 3.4. Homologous Recombination

**Figure 5:**
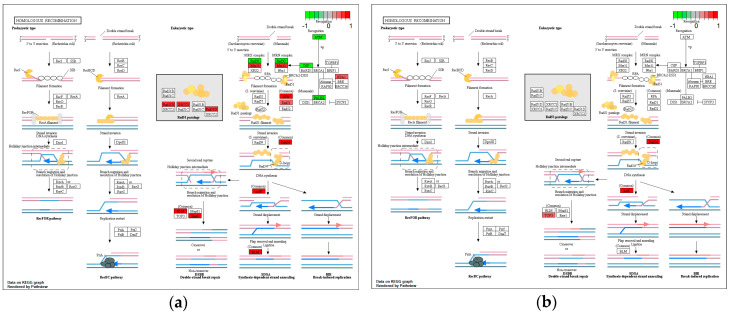
Impact of *H. pylori* infection (**a**) and CagA protein (**b**) on gene expression of homologous recombination (HR) components, visualized in KEGG pathway map. Components involved in the process are highlighted in red (downregulated) or green (upregulated).

Of the 41 genes involved in HR, *H. pylori* infection appeared to deregulate 19 genes, of which four were found upregulated, namely, *ATM*, *RAD50*, and *CTIP*, which are involved in damage recognition and response, and *PALB2*, which is involved in homology searching and strand invasion (Figure 5a). On the other hand, 15 genes were found downregulated, such as *MRE11A (MRE11)*, *RPA1*, *RPA2*, *RPA3*, *XRCC3*, *BABAM1*, *RAD51D*, and *RAD51* involved in damage recognition and processes both preceding and mediating strand invasion, and *RAD54B*, *RAD54L (RAD54)*, *POLD1*, *POLD2*, *POLD3*, *EME1*, and *BLM*, involved in post-strand invasion, as well as DNA synthesis and recombination processes (Figure 5a). Expression of CagA appeared to contribute to the downregulation of four of the aforementioned genes (*RAD54L*, *POLD1*, *POLD2*, and *POLD3*) and, paradoxically, of *TOP3A*, which was not observed downregulated as a result of the infection (Figure 5b).

### 3.5. Non-Homologous End-Joining

**Figure 6:**
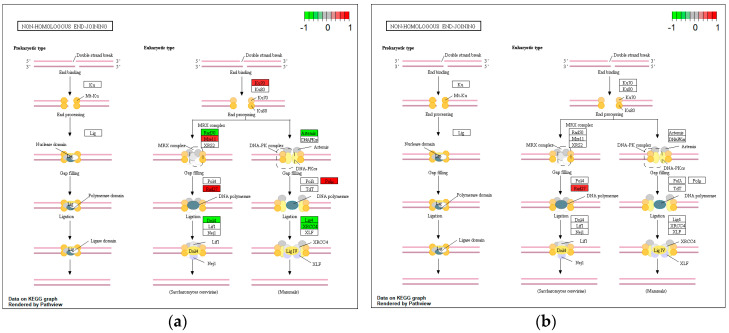
Impact of *H. pylori* infection (**a**) and CagA protein (**b**) on gene expression of non-homologous end-joining (NHEJ) components, visualized in KEGG pathway map. Components involved in the process are highlighted in red (downregulated) or green (upregulated).

With reference to the NHEJ, *H. pylori* infection was found to downregulate damage recognizing components *KU70* and *MRE11*, as well as gap filling and end-processing components *POLM* and *FEN1 (RAD27)* (Figure 6a), the latter being the only component predicted to also be downregulated in a CagA-related manner (Figure 6b). At the same time, upregulation of key components that catalyze end-processing and ligation, namely, *RAD50, ARTEMIS*, *XRCC4*, and *LIG4*, was observed upon infection (Figure 6a).

### 3.6. Key DNA Damage Repair Component Deregulation at the Protein Level

**Figure 7:**
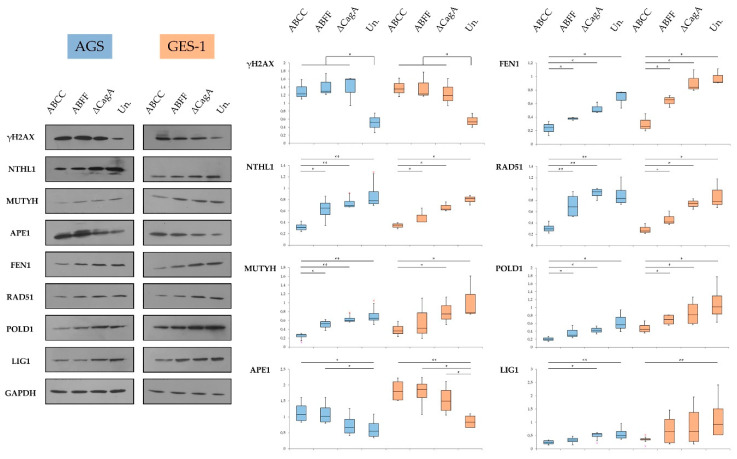
Expression of DNA damage repair components in *H. pylori*-infected AGS (blue) and GES-1 (orange) cells. Results suggest CagA-independent increase of Ser139 phosphorylated histone H2AX (γH2AX) and decrease of Nth Like DNA Glycosylase 1 (NTHL1), MutY DNA Glycosylase (MUTYH), Flap Structure-Specific Endonuclease 1 (FEN1), RAD51 Recombinase, DNA Polymerase Delta Catalytic Subunit (POLD1), and DNA Ligase 1 (LIG1) protein levels, related to the expression and phosphorylation of CagA. Apurinic/Apyrimidinic Endodeoxyribonuclease 1 (APE1) protein levels were increased during the infection in a CagA-related manner. Quantification of protein levels was conducted by densitometry in at least three experimental replicates per condition. Statistical analysis was performed using Mann–Whitney *U* test (levels of significance: + *p* = 0.1–0.05, * *p* = 0.05–0.01, ** *p* < 0.01); Un.: uninfected control.

Following transcriptome analysis and the identification of putative deregulated DDR genes as a result of *H. pylori* infection and CagA expression and phosphorylation status (Figure 1), we performed validation at the protein expression level utilizing AGS and GES-1 cell lines. We observed an increase in γH2AX upon infection, in both cell lines, regardless of the expression and phosphorylation of CagA protein (Figure 7). The protein levels of NTHL1, MUTYH, FEN1, RAD51, POLD1, and LIG1 were observed to be decreased, during the infection, in a CagA- and phospho-CagA-related manner in both cell lines (Figure 7). The levels of APE1 protein were found consistently increased during *H. pylori* infection, and CagA expression appeared to contribute to this upregulation (Figure 7).

## 4. Discussion

In 1994, the World Health Organization characterized *H. pylori* as a class I human carcinogen [2], identifying the induction of chronic active inflammatory response and oxidative stress, and the deregulation of apoptosis and cellular proliferation as the fundamental processes promoting carcinogenesis and highlighting CagA as a major virulence factor [3]. More recently, *H. pylori* has also been implicated as a crucial contributor to gastric carcinogenesis via modulation of DNA damage repair and response [44], although the underlying mechanisms have not been fully clarified.

Our transcriptome analysis revealed that 5227 genes were deregulated during *H. pylori* infection, of which 657 appeared to be related to CagA expression and 21 were associated with CagA phosphorylation. Pathway enrichment analysis suggested that DDR mechanisms were significantly affected by the infection and CagA was an important factor of their deregulation, with the exception of NHEJ. Most specifically, we documented an attenuation of BER and MMR and a more intricate deregulation of NER, HR, and NHEJ. Our transcriptome data obtained are in line with those reported by others utilizing different *cag*PAI-positive strains [27].

The majority of the genes involved in BER were observed to be downregulated. BER is responsible for repair of damaged DNA bases (such as products of oxidation), base mismatches, AP-sites, and single-strand breaks (SSBs) [43,66,67,68]. In this respect, BER is a critical mechanism for repair of the RONS-induced DNA damage during *H. pylori* infection [69]. During BER, the damaged bases are recognized and removed by DNA glycosylases, including *NTHL1*, *NEIL2*, *NEIL3*, *UNG*, *SMUG1*, *MUTYH*, and *MPG*, which, in our study, were found to be downregulated, potentially explaining the increase in point mutations in nuclear and mitochondrial DNA, reported in *H. pylori* infection [35,36,40]. Regarding NEIL2, our observations are in agreement with a recent report [70]. Transcriptional downregulation of *UNG* and *MUTYH* was observed to be related to CagA expression. Furthermore, we observed that expression of CagA can contribute to the reduction of MUTYH and NTHL1 protein levels, thereby affecting damage recognition and downstream repair. *MBD4* glycosylase was found upregulated in a CagA-independent manner, which could be a direct effect of the infection on gene expression or an indirect response related to the emergence of DNA lesions. OGG1 glycosylase is responsible for the repair of 8-oxo-G, a characteristic oxidative damage promoted by *H. pylori* infection [24], and, in agreement with our data, its expression has been reported to be unaffected during infection [35,70]. Glycosylases play a crucial role in the repair of oxidative base lesions preventing genomic instability and carcinogenesis [69,71,72]. OGG1 removes oxidative purine lesions, while MUTYH prevents G:C→T:A transversions by removing misincorporated A from A:8-oxo-G base pairs, thus allowing OGG1 to repair the lesion [73]. NTHL1 removes oxidized pyrimidine lesions [67], thereby preventing DNA synthesis blocking and averting G:C→T:A and C:G→G:C transversions, C:G→T:A transitions, and miscoding [74,75,76,77]. *MUTYH* and *NTHL1* defects are associated with increased risk for cancer, and C:G→A:T transversions and C:G→T:A transitions are respectively associated with MUTYH- and NTHL1-related tumors [78,79,80,81,82]. Shimizu et al. reported that such transitions are the predominant mutations in *H. pylori*-induced inflammation of gastric mucosa and tumors [31], whereas Touati et al. reported mainly transversions including G:C→T:A, A:T→C:G, and A:T→T:A in a mouse model [36]. The infection can also enhance mitochondrial DNA (mtDNA) mutations, mainly transitions (A:T→G:C and G:C→A:T) which are related to bacterial virulence factors including CagA [35,40]. Moreover, *H. pylori* infection and CagA protein have been described to induce *TP53* mutations in gastric tumors, mainly insertions/deletions and transitions [38,83].

In our study, *APE1* was observed to be transcriptionally downregulated during the infection, in a CagA-related manner, while APE1 protein levels were found consistently increased. Machado et al. also reported the transcriptional downregulation of *APE1* during the infection [35], however, other reports supported the overexpression of APE1 transcript and protein levels [52,53]. These seemingly contradictory observations could be explained by differences in experimental conditions, such as length of infection or MOI used, as well as by promotion of a putative negative feedback loop. Consequently, a more detailed investigation of APE1 transcription at various time-points is warranted. Potential APE1 downregulation and glycosylase upregulation (e.g., MBD4) or constant expression (e.g., TDG, NEIL1, OGG1) can generate an imbalance between AP-site formation and repair that can lead to accumulation of AP-sites, point mutations, and DNA breaks. On the other hand, an increase in APE1 could result in the accumulation of nicks, which, if combined with downregulation of downstream BER components, can promote formation of DNA breaks. In any case, APE1 seems to fulfil an important role during infection by regulating genomic stability, oxidative stress, and inflammation, thus affecting the risk of carcinogenesis [84,85,86,87,88,89,90]. Our results show that the infection can also downregulate *APE2* [91,92], in a CagA-independent manner, which could potentially modulate the response to oxidative stress and SSBs by compromising the ATR/ Checkpoint Kinase 1 (CHK1) pathway [93]. Interestingly, studies have reported decreased activation of ATR and ATRIP during *H. pylori* infection [30].

With respect to subsequent stages in the BER process, we observed a downregulation in the genes encoding FEN1, PCNA, POLD, POLE, LIG1, and LIG3. Downregulation of these genes can impair DNA synthesis and repair and potentially promote replication stress, a condition that can introduce DSBs. CagA protein appeared to be involved in the downregulation of *FEN1*, *POLD1*, *POLD2*, *POLD3*, *POLE*, and *LIG1*. The downregulation of *POLD3* has been correlated with miR-150-5p deregulation during *H. pylori* infection [50]. In addition, we documented CagA-independent downregulation of *PARP1* and upregulation of *PARP4*. Poly(ADP-Ribose) Polymerases (PARPs) are a family of polymerases involved in DDR, genomic integrity, and programmed cell death [94], and their main role is to detect and mediate the repair of SSBs. In this respect, PARPs downregulation could affect damage repair, and their upregulation could be a sign of damage introduction or apoptosis.

FEN1 catalyzes the removal of the flaps generated during DNA replication and long-patch BER (LP-BER), and it is involved in hairpin structure removal and resolution of stalled replication forks [95]. FEN1 also maintains the integrity of telomeres and GC-rich repeat DNA sequences [96,97]. In this respect, FEN1 deficiency can induce mutations, chromosomal instability, and mini- and micro-satellite instability, thereby predisposing to cancer development [98,99,100,101,102]. In our infection system, CagA expression was observed to promote decrease of FEN1, thereby potentially favoring genomic instability. *H. pylori* infection has been reported to introduce DNA strand breaks [23,26,27,30] and MSI in both nuclear and mitochondrial DNA [35,37,39]. In addition, the infection has been reported to induce telomere length shortening in the gastric mucosa, which is related to inflammation-induced oxidative DNA damage [103]. FEN1 mediates replication in telomere regions, and its decrease could result in replication fork stalling and DNA breaks. Adding to that, it is interesting to mention that *H. pylori* infection is reported to accumulate DSBs in telomere-proximal regions. Therefore, infection-induced FEN1 decrease could also be a contributing factor to telomere loss and chromosomal instability. Our study showed that infection can promote an imbalance between APE1 and the downstream proteins of LP-BER FEN1, POLD1, and LIG1. This imbalance could result to the introduction and the attenuated downstream repair of AP-sites that could promote replication stress and formation of BER intermediates, which can ultimately introduce DSBs and genomic instability.

Our data support that *H. pylori* infection can decrease POLD1 and LIG1 levels, in a CagA-related manner. *POLD1* encodes the catalytic subunit of POLD which performs high-fidelity DNA synthesis, and its participation in multiple repair pathways highlights its crucial role in the genomic stability [104]. A decrease in POLD1 levels has been reported to result in accumulation of DNA damage during oxidative stress [105], and POLD1 defects can result in accumulation of point and frameshift mutations and DSB introduction via fork collapse, thus predisposing to carcinogenesis [104,106,107,108]. Furthermore, *POLE* and *POLD1* defects have been reported to be instrumental in the development of cancer, characterized by increased levels of transversions (G:C→T:A and A:T→C:G) and transitions (G:C→A:T) [106,109]. LIG1 catalyzes proper ligation during LP-BER, MMR, NER, and discontinuous DNA replication, thereby preventing the formation of strand breaks [110]. Its crucial role is also accentuated by the fact that LIG1 deficiency can promote hypersensitivity to DNA damaging [111], accumulation of strand breaks [112], replication errors, genomic instability, and increased risk for carcinogenesis [110]. The decrease in POLD1 and LIG1 levels can modulate the precision of DNA synthesis and DDR, and thereby could potentially contribute in the aforementioned increase of point and frameshift mutations that are reported during the infection, as well as favoring the promotion of replication stress and strand breaks.

*H. pylori* infection induces oxidative stress, which can lead to the formation of bulky DNA adducts, potentially hampering DNA replication and transcription [22]. These lesions can be repaired through NER [113] and, in our study, *H. pylori* infection has been shown to deregulate the expression of 24 out of 47 genes involved in this mechanism. Our results supported the CagA-independent upregulation of *XPC*, *CUL4B*, and *ERCC6* which are critical elements in damage recognition, an observation possibly linked to DNA damage introduction. Interestingly, other NER genes involved in damage recognition, such as *DDB1*, *CETN2*, and *RBX1*, were found to be downregulated during the infection and, in the case of *DDB1*, data support a putative involvement of CagA. The infection was also observed to affect the next stage of NER, by deregulating Transcription Factor II H (TFIIH) complex components. Particularly, *XPD*, which is critical for DNA unwinding at the damaged foci, displayed CagA-related downregulation, while *CDK7* and *CCNH* showed CagA-independent upregulation. In addition, the *RPA* subunits *RPA1*, *RPA2*, and *RPA3* were observed to be downregulated in a CagA-independent manner, resulting in limited protection of single stranded DNA, potentially favoring formation of secondary structures and inefficient DNA unwinding, thus affecting the fidelity of the repair process. Regarding the stage of nick insertion on lesion site, our data suggest that *XPF* showed CagA-independent upregulation. Interestingly, XPG and XPF endonucleases have been reported to be involved in the expression of NF-κB target genes and DSB introduction, in a *cag*PAI-dependent manner, during *H. pylori* infection [28]. NER concludes with DNA synthesis and ligation via POLD, POLE, and LIG1, assisted by PCNA and RFC. Our data suggest that the accessory components (*RFC2*, *RFC3*, *RFC5*, *PCNA*), polymerases (*POLD1*, *POLD2*, *POLD3*, *POLE*, *POLE2*, *POLE4*), and ligase *LIG1*, were downregulated during infection, for which the downregulation of *RFC5, POLD1*, *POLD2*, *POLD3*, *POLE*, and *LIG1* was CagA-related. This modulation could lead to DDR impairment and replication stress, thus favoring genomic instability.

MMR is responsible for the repair of base mismatches and prevention of MSI [114]. Our data suggest that *H. pylori* infection can attenuate this mechanism via downregulation of 14 out of 23 genes involved in MMR. The infection seems to downregulate, in a CagA-independent manner, core components *MSH2* and *MSH6* involved in damage recognition and *MLH1* which is involved in nick insertion. Previous studies support the aforementioned observations [35,49], and, in line with those findings, *MLH1* has been suggested to be silenced via promoter hypermethylation [115,116], while *MSH2* and *MSH3* have been reported to be downregulated via deregulation of miR-155-5p and miR-3163, respectively [50]. As mentioned earlier, we observed downregulation of genes encoding accessory factors PCNA and RFC, which support the activity of MMR components MutS and MutL, involved in damage recognition, nick insertion, and subsequent DNA synthesis. Therefore, their decreased expression could contribute to diminished DNA damage recognition and nick insertion, as well as impaired DNA synthesis and ligation. As mentioned earlier, *RPA1*, *RPA2*, *RPA3*, *POLD1*, *POLD2*, *POLD3*, and *LIG1* were observed to be downregulated, of which the polymerases and ligase were downregulated in a CagA-related manner. Decreased levels of RPA, POLD, and LIG1 can lead to promotion of replication stress and genomic instability.

HR is the high-fidelity mechanism for the repair of DSBs, utilizing homologous sequences as a template [117]. Our study reported the deregulation of 19 out of 41 genes of this mechanism during *H. pylori* infection. We observed that 24 h post infection, *MRE11* was found downregulated, whereas *RAD50*, *CTIP*, and *ATM* were found upregulated in a CagA-independent manner. Hanada et al. also reported increased gene expression and activation of ATM [27]. The observed upregulation of *ATM*, *CTIP*, and *RAD50* could indicate a putative cellular response in the introduction of DSBs. Potential MRE11 downregulation could impact the MRE11–RAD50–NBS1 complex-mediated DSB recognition and response initiation. RAD51 has a central role in HR, mediating strand invasion and homology searching, and its deficiency has been reported to lead to accumulation of cells in G2/M phase, promotion of chromosomal instability, and carcinogenesis [118,119,120]. Our results regarding the CagA-related decrease of RAD51 protein and *RAD54L*, during *H. pylori* infection, are consistent with Hanada et al. [27]. In line with this, a recent report also suggested the CagA-related RAD51 decrease, documenting the deregulation of the SNHG17/miR-3909/RING1/RAD51 and SNHG17/NONO pathways, thereby attenuating HR and favoring the repair of DSBs via the error-prone NHEJ [121]. Decreased expression of POLD, RPA, RAD51, and RAD54L can result in diminished performance of HR, promotion of replication stress, and, therefore, increased DSB introduction [122]. EME1 and BLM were observed to be downregulated in a CagA-independent manner, which could affect processes involved in the resolution of Holliday junctions, DSB end resection, and displacement of the invading strand.

The aforementioned reports illustrate the attenuation of HR and support the shift of DSB repair toward NHEJ [123]. Indeed, NHEJ has been shown to be the predominant mechanism for DSB repair during *H. pylori* infection [28]. Our study supports that infection promotes CagA-independent downregulation of *KU70*, which potentially results in decreased capacity of damage recognition and NHEJ initiation and, therefore, increased DSB formation. In addition, our study also suggests the CagA-independent upregulation of key NHEJ components, namely, *LIG4*, *XRCC4*, and *ARTEMIS*, which could be explained as a response to the introduction of DSBs. Furthermore, *POLM* was observed to be downregulated during infection, possibly affecting the processing of DSBs ends and the implementation of end-joining.

Lastly, in an attempt to correlate the observed downregulation of DDR components with the resultant DNA damage outcome, we investigated the levels of γH2AX, a characteristic marker of DSBs. We observed that γH2AX levels were elevated during *H. pylori* infection regardless of the presence and phosphorylation of CagA. Toller et al. reported that the infection induces DSBs via direct bacterial adhesion, in a *cag*PAI-dispensable manner, and prolonged infection seemed to result in DSBs accumulation, potentially through *H. pylori*-promoted DDR saturation [26]. Koeppel et al. supported that infection with *cag*PAI-positive *H. pylori* strains can induce the formation of DSBs which are accumulated in regions transcribed and proximal to telomeres, raising the point that the particular DNA damage pattern was consistent with genomic aberrations observed in gastric adenocarcinomas [30]. In addition, *H. pylori* infection has been reported to induce DSBs regardless of the presence of *cag*PAI, yet *cag*PAI and CagA were suggested to contribute to higher levels of DSBs [27,30]. In contrast, in our experimental layout, we did not observe significant differences in γH2AX levels between the CagA-expressing and ΔCagA strains. Hartung et al. also highlighted the role of *cag*PAI in DSB introduction, via T4SS binding in gastric epithelial cells and consequent activation of NF-κB signaling [28]. However, as all the strains in our experimental design were *cag*PAI-positive, we cannot draw any conclusion about *cag*PAI involvement in DSB formation, as well as DDR modulation. Despite the fact that we observed a CagA-related increase in APE1 levels and a simultaneous decrease in DDR proteins such as RAD51, FEN1, POLD1, and LIG1, which could contribute to some degree to the formation of DSBs, we did not observe a respective increase in γH2AX levels, indicating that additional complex mechanisms determine the formation of DNA breaks.

## 5. Conclusions

*H. pylori* infection induces a plethora of DNA damages on gastric epithelial cells, necessitating an integrous DDR machinery in order to prevent the promotion of genomic instability. Our study suggests that *H. pylori* infection and its major virulence factor CagA can lead to decreased levels of critical DDR components, thereby compromising the capacity of the DDR machinery. This modulation can potentially disrupt the balance between DNA damage introduction and repair in gastric epithelial cells, thus favoring genomic instability and contributing to gastric cancer development.

## Figures and Tables

**Table 1:** Pathway enrichment analysis with regards to DNA damage repair (DDR) mechanisms.

		*Helicobacter pylori* Infection		CagA	
DDR Mechanism	*N* ^1^	DEGs ^2^	FDR	DEGs ^2^	FDR
BER	33	24	2.7 × 10^−7^	9	1.2 × 10^−5^
NER	47	24	9.2 × 10^−4^	8	8.4 × 10^−4^
MMR	23	14	1.1 × 10^−3^	5	2.4 × 10^−3^
HR	41	19	7.5 × 10^−3^	5	2.3 × 10^−2^
NHEJ	13	8	9.2 × 10^−3^	1	3.9 × 10^−1^

^1^*N*: Total number of genes involved in the corresponding mechanism. ^2^ DEGs: Differentially expressed genes deregulated in the corresponding mechanism.
